# Moderate amplifications of the c-myc gene correlate with molecular and clinicopathological parameters in colorectal cancer.

**DOI:** 10.1038/bjc.1998.390

**Published:** 1998-06

**Authors:** L. Masramon, R. Arribas, S. TÃ³rtola, M. Perucho, M. A. Peinado

**Affiliations:** Institut de Recerca OncolÃ²gica (Department of Cancer and Metastasis), Hospital Duran i Reynals, Barcelona, Spain.

## Abstract

**Images:**


British Journal of Cancer (1998) 99(12), 2349–2356
© 1998 Cancer Research Campaign

### Moderate amplifications of the c-*myc* gene correlate with molecular and clinicopathological parameters in colorectal cancer

**L Masramon[1], R Arribas[1], S Tórtola[1], M Perucho[2] and MA Peinado[1]**

[1]Institut de Recerca Oncològica (Department of Cancer and Metastasis), Hospital Duran i Reynals, Autovia Castelldefels km 2.7, L'Hospitalet, 08907 Barcelona, Spain; [2]The Burnham Institute, 10901 North Torrey Pines Rd, La Jolla 92037 CA, USA

**Summary** C-*myc* gene activation is a common event in multiple types of neoplasia and has been associated with different cellular processes relevant to the malignant transformation of cancer cells. C-*myc* gene amplification has been analysed in colorectal carcinomas by means of an innovative DNA fingerprinting method based on the arbitrarily primed PCR. This method requires a low amount of DNA, uses multiple internal controls and appears sensitive and reproducible. Clinicopathological and molecular correlates have been investigated in a series of 70 colorectal carcinomas. The incidence of c-*myc* amplification was 26%, ranging from two- to fivefold increase in copy number. C-*myc* amplification occurrence was more frequent in more advanced stages of tumour invasion ($P < 0.001$) and was associated with mutations in the *p53* tumour-suppressor gene ($P = 0.048$). The presence of c-*myc* amplification was indicative of a shorter disease-free survival period but, because of its strong association with Dukes' stage, its prognostic value is questionable.

**Keywords:** oncogene amplification; tumour progression; genetic alteration; arbitrarily primed PCR; DNA fingerprinting

After the initial finding of an amplified c-*myc* gene (also known as MYC) in different human cell lines (Collins and Groudine, 1982; Alitalo et al, 1983; Little et al, 1983), oncogenic activation of the c-*myc* gene through amplification has been demonstrated in multiple types of cancer (reviewed by Bishop, 1991; Garte, 1993). Previous studies have shown that c-*myc* gene amplification and/or overexpression in colorectal cancer correlates with the degree of invasion (Kozma et al, 1994; Sato et al, 1994) and poor differentiation (Heerdt et al, 1991). Nevertheless, other authors have failed to find such associations (Erisman et al, 1988; Finley et al, 1989; Matsumara et al, 1990; Nagai et al, 1992; Smith et al, 1993). Although c-*myc* overexpression appears in an elevated proportion of tumours (66–90%) (Erisman et al, 1985; 1988; Matsumara et al, 1990; Smith et al, 1993; Sato et al, 1994; Wang et al, 1994), gene amplification, as determined by Southern (Erisman et al, 1985; Untawale and Blick, 1988; Finley et al, 1989; Matsumara et al, 1990; Heerdt et al, 1991) or dot-blot hybridization (Erisman et al, 1988; Nagai et al, 1992; Smith et al, 1993; Kozma et al, 1994; Wang et al, 1994) is less frequent, ranging from 0 to 30%. In addition to the intrinsic complexity of cellular processes, sample heterogeneity and methodological pitfalls are important factors that puzzle the comprehensibility of these divergences.

With the advent of the polymerase chain reaction (PCR) and in order to overcome the limitation of blot hybridization techniques (mainly, a high amount of genomic DNA is required), different quantitative PCR methods for c-*myc* analysis have been developed (Rhoer-Moja et al, 1993; Watson et al, 1993; Sugimoto et al, 1994; Sestini et al, 1995). PCR co-amplification of the c-*myc* gene with an internal control did not render reliable results in our hands. Theoretical and practical considerations limit the accuracy of this type of technique, often generating irreproducible results (see review by Ferré et al, 1994).

The initial aim of this study was to determine the possible relationship between c-*myc* gene amplification and molecular and clinicopathological variables in a series of colorectal carcinomas. As only a limited amount of DNA was available from normal and tumour tissue (making the use of hybridization-type methods impossible) and in order to overcome the technical limitations described above, we have set up a PCR-derived method that uses multiple internal and arbitrary controls. This technique is based on the arbitrarily primed PCR (AP-PCR) (Welsh and McClelland, 1990), a fingerprinting-type technique using primers whose nucleotide sequence is arbitrarily chosen.

It has been demonstrated that AP-PCR is useful for the detection and isolation of tumour-specific allelic losses and gains (Peinado et al, 1992; Kohno et al, 1994; Achille et al, 1996), thus providing a molecular alternative to cancer cytogenetics. In addition, DNA fingerprinting by AP-PCR permitted the discovery of ubiquitous somatic genomic instability in a subset of colorectal tumours (Ionov et al, 1993). More recently, AP-PCR fingerprinting has been used to estimate the degree of genomic damage in colorectal tumours (Bocker et al, 1996; Arribas et al, 1997; Basik et al, 1997). We have observed that the rate of genomic damage in neoplastic cells, as determined by AP-PCR, may have important prognostic applications (Arribas et al, 1997). Two intrinsic features of AP-PCR are of special interest: (a) the amplified sequences proceed from randomly selected genome regions with no apparent bias for the chromosomal origin of the bands (Peinado et al, 1992; Yasuda et al, 1996); and (b) the amplification is quantitative in that the intensity of an amplified band is proportional to the concentration of its corresponding template sequence (Peinado et al, 1992; Perucho et al, 1995).

*Received 9 September 1997*
*Revised 5 November 1997*
*Accepted 12 November 1997*

*Correspondence to:* MA Peinado

In our approach, primers are designed to amplify a fragment including the third exon of the human c-*myc* gene and PCR is performed under low-stringency conditions, as in AP-PCR. In consequence, the c-*myc* gene fragment is co-amplified with a number of arbitrary sequences that consistently appear in all the samples. These sequences are flanked by the same primers as the c-*myc* gene and competition among them generates a quantitative fingerprint (see scheme in Figure 1). Furthermore, we can expect

**Table 1** Molecular and clinical data of patients classified by the Dukes' stage[a]

| Stage | Case | Age | Sex | DFS | Rec | Follow-up (months) | Dead | *p53* | *ras* | *MMP* | c-*myc* |
|---|---|---|---|---|---|---|---|---|---|---|---|
| Dukes | 6 | 77 | M | 62 | Yes | 94 | No | + | – | – | – |
| A | 44 | 77 | F | 96 | No | 96 | No | + | – | – | – |
| | 51 | 67 | F | 68 | Yes | 68 | No | – | – | + | – |
| | 57 | 71 | M | 83 | No | 83 | No | + | + | – | – |
| | 58 | 69 | M | 78 | No | 78 | No | – | + | – | – |
| | 60 | 75 | F | 43 | No | 43 | No | ND | – | – | – |
| | 66 | 76 | F | 44 | Yes | 80 | No | + | – | – | – |
| | 83 | 73 | M | 52 | No | 52 | No | – | – | – | – |
| | 89 | 39 | M | 84 | No | 84 | No | – | – | + | – |
| | 119 | 61 | M | 60 | No | 60 | No | + | – | – | – |
| | 135 | 79 | F | 65 | No | 65 | No | – | + | – | + |
| | 180 | 55 | M | 81 | No | 81 | No | + | – | – | – |
| Dukes | 33 | 61 | M | 29 | Yes | 29 | Yes | + | – | – | – |
| B | 36 | 80 | F | 27 | Yes | 27 | Yes | + | + | – | – |
| | 42 | 77 | M | 85 | No | 85 | No | + | + | – | – |
| | 45 | 76 | M | 84 | Yes | 84 | No | ND | + | – | – |
| | 47 | 59 | M | 87 | Yes | 87 | No | + | + | – | – |
| | 48 | 81 | M | 31 | Yes | 31 | Yes | – | + | – | – |
| | 49 | 60 | M | 62 | Yes | 84 | Yes | – | – | – | – |
| | 55 | 68 | M | 84 | Yes | 84 | No | – | + | – | – |
| | 59 | 56 | M | 70 | No | 70 | No | – | + | – | – |
| | 62 | 62 | M | 79 | No | 79 | No | + | – | – | – |
| | 68 | 73 | M | 34 | Yes | 34 | No | – | + | + | – |
| | 78 | 62 | M | 71 | Yes | 71 | No | + | – | – | + |
| | 91 | 53 | F | 28 | Yes | 28 | No | ND | – | + | – |
| | 100 | 60 | F | 61 | No | 61 | No | ND | + | – | – |
| | 118 | 70 | M | 61 | No | 61 | No | + | – | – | – |
| | 121 | 66 | F | 45 | Yes | 45 | Yes | + | – | – | + |
| | 132 | 71 | M | 59 | No | 59 | No | – | – | + | – |
| | 136 | 58 | M | 19 | Yes | 19 | Yes | + | – | – | – |
| | 150 | 69 | M | 15 | Yes | 15 | Yes | + | + | – | – |
| | 153 | 63 | M | 49 | No | 49 | No | + | – | – | + |
| | 188 | 56 | M | 44 | Yes | 46 | Yes | – | + | – | – |
| | 190 | 63 | M | 68 | No | 68 | No | – | – | – | – |
| | 193 | 68 | F | 48 | No | 48 | No | + | – | – | – |
| Dukes | 27 | 63 | M | 5 | Yes | 5 | Yes | + | – | – | + |
| C | 56 | 76 | M | 84 | No | 84 | No | + | – | – | – |
| | 94 | 56 | M | 67 | Yes | 67 | No | + | – | – | + |
| | 96 | 66 | M | 20 | Yes | 20 | Yes | – | + | – | – |
| | 101 | 61 | M | 61 | No | 61 | No | + | – | – | – |
| | 117 | 93 | M | 9 | Yes | 9 | Yes | + | + | – | – |
| | 134 | 62 | F | 66 | No | 66 | No | + | – | – | + |
| | 137 | 71 | M | 29 | Yes | 29 | Yes | + | + | – | + |
| | 156 | 60 | M | 30 | Yes | 57 | Yes | ND | – | – | – |
| | 189 | 71 | F | 6 | Yes | 6 | Yes | + | – | – | – |
| | 191 | 78 | M | 3 | Yes | 3 | Yes | – | – | – | + |
| | 192 | 64 | M | 78 | Yes | 78 | No | + | – | – | + |
| | 201 | 54 | F | 76 | No | 76 | No | – | – | + | – |
| | 202 | 53 | M | 69 | No | 69 | No | + | – | – | + |
| | 213 | 48 | M | 22 | No | 22 | No | – | – | – | – |
| Dukes | 65 | 64 | M | 52 | Yes | 52 | Yes | – | + | – | + |
| D | 80 | 57 | M | 9 | Yes | 9 | Yes | ND | – | – | + |
| | 125 | 56 | F | 12 | Yes | 12 | Yes | + | – | – | + |
| | 133 | 47 | M | 21 | Yes | 21 | Yes | + | + | – | – |
| | 182 | 69 | F | 36 | Yes | 36 | Yes | + | – | – | – |
| | 197 | 57 | M | 5 | Yes | 5 | No | – | – | + | – |
| | 268 | 77 | M | 5 | Yes | 5 | Yes | + | – | – | + |

[a]Only cases with available follow-up data are included. Sex: M, male, F, female; DFS, disease-free survival in months; Rec, recurrent disease; Dead: Yes, died of disease; No, alive with or without disease, dead of unrelated causes; *p53* and *ras*: +, with mutation; – no mutation; ND, not determined; MMP, microsatellite mutator phenotype: +, instability in at least two out of five microsatellites sequences; –, no instability; c-*myc*; +, gene amplification; –, no gene amplification.

 *© Cancer Research Campaign 1998*

that these sequences are of different chromosomal origin and, in consequence, they are useful as internal controls. As the rationale of this technique is based on the properties of AP-PCR, we have called this method targeted AP-PCR (TAP-PCR).

We have applied TAP-PCR to the allelic dosage of the c-*myc* gene in a series of colorectal tumours of which a limited amount of DNA was available. We have observed moderate c-*myc* gene amplifications in about one-quarter of the tumours. Amplifications of the c-*myc* gene occurred more frequently in advanced stages of tumour invasion and were associated with mutations in the *p53* gene.

### MATERIAL AND METHODS

### Samples

Seventy colorectal carcinomas and paired normal tissue samples were obtained from the Human Tissue Cooperative Network (University of Alabama, Birmingham, USA). Phenotypic and genetic characteristics of these cases have been described elsewhere as part of a larger series (Capella et al, 1991; Peinado et al, 1993). The samples used in this study were selected from a collection of 181 cases based on availability of genomic DNA from tumour and paired normal mucosa. They were representative of the whole collection for all the molecular and clinicopathological parameters considered. Cases were pathologically staged using Astler–Coller modification of Dukes' classification system. Detailed histological study of a short series of cases revealed that in most cases (16 out of 17) more than 75% of the analysed tissue was composed of neoplastic cells. In a single case, stromal and normal epithelial cells constituted approximately 50% of the tissue. SW480 and DLD-1 cell lines (ATCC, Rockville, MD, USA) were used as positive and negative controls, respectively, for c-*myc* amplification. Cases with perioperative deaths and with insufficient follow-up were excluded from the survival analysis.

### Molecular analyses

Genomic DNA was extracted by the phenol–chloroform method as described previously (Nakano et al, 1984). DNA was diluted to a concentration of 20 ng $\mu l^{-1}$ and 1 µl of each DNA was run in a 0.75% agarose gel and stained with ethidium bromide to verify its quality and concentration. When necessary, the DNA concentration was adjusted according to the ethidium bromide signal.

Mutations at codons 12 and 13 of the K-*ras* gene and 12 of the N-*ras* gene were detected and characterized by the artifical RFLP/PCR approach (Capella et al, 1991). *p53* mutations in exons 4–9 were analysed using single-strand conformation polymorphism (SSCP) of PCR amplified products and characterized by direct cycle sequencing of the PCR product (Peinado et al, 1993). Genomic instability at simple repeated sequences was analysed in microsatellite sequences AP2, AP3, Mfd27, Mfd41 and Mfd47 as described previously (Ionov et al, 1993; Shibata et al, 1994). Cases showing instability in two or more microsatellites were considered to belong to the microsatellite mutator phenotype (MMP) pathway. In agreement with other reports (Thibodeau et al, 1993), tumours with low genomic instability (mutations in only one out of five microsatellite sequences analysed) present molecular and biological characteristics different from those with high instability but similar to the rest. In consequence, they were considered MMP negative.

### TAP-PCR of c-*myc*

TAP-PCR reactions were performed in duplicate with 50 ng of genomic DNA, 125 µM each dNTP, 1 µM each primer (sense: 5′-AAAGAGGCAGGCTCCTGGCA-3′, antisense: 5′-TCTCGTCGTTTCGCAACAA-3′), 1 µCi [$\alpha$-$^{33}$P]dATP (Amersham, Buckinghamshire, UK) and 1.25 units of *Taq* polymerase (Boehringer Mannheim, Mannheim, Germany) in PCR buffer (10 mM Tris-HCl pH 8.0, 50 mM potassium chloride, 1.5 mM magnesium chloride) in a final volume of 25 µl. The reaction consisted of five low-stringency cycles (30 s at 94°C, 30 s at 50°C, 30 s at 72°C) and 35 high-stringency cycles (30 s at 94°C, 30 s at 65°C, 30 s at 72°C) and was carried out in a PTC-100 thermocycler (MJ Research, Watertown, MA, USA). The product was diluted with formamide dye buffer, denatured for 3 min at 95°C and 3 µl were run on a 6% acrylamide 8 M urea sequencing gel at 55 W for 3 h. The gels were dried under vacuum at 85°C and exposed to radiograph film at room temperature without intensifier screen for 1–3 days. The amplified c-*myc* region corresponds to a fragment 470-bp-long, which includes

**Figure 1** Principles of TAP-PCR method. Conventional PCR amplification using specific primers (**A**) will produce a unique product corresponding to the sequence flanked by the primers and resolved as a unique band by gel electrophoresis (lane A). When low stringency conditions are applied, primers are likely to anneal to multiple regions of the genome (**B** and **C**) and many sequences are co-amplified in a competitive and reproducible fashion, including the one for which the primers are designed (lanes B and C). In this case, differences in copy number of the targeted sequence will be manifested as proportional changes in the amount of its corresponding TAP-PCR product band as resolved by electrophoresis. Right panel corresponds to a 6% polyacrylamide (non-denaturing) gel electrophoresis of c-*myc* amplification in the conditions described above. Lanes B and C contain the TAP-PCR products from a normal and its paired tumour DNA respectively. Gel was stained with ethidium bromide and colour inverted

© Cancer Research Campaign 1998

**Figure 2** c-*myc* allelic dosage of SW480 and DLD-1 cell line DNAs. (Top) Duplicated TAP-PCR analysis of pure and mixed DNAs (mixture proportions are indicated at top) from these two cell lines. Solid arrow indicates the band identified as c-*myc*. Differences of intensity in this band are observed according to the mixture proportions as shown in the densitometric analysis at right. For calculation purposes, the intensity of five arbitrary bands (indicated by empty arrows) was monitored and used as reference. Densitogram is displayed for lanes marked with an asterisk. (Bottom) Southern blot hybridization analysis of the same cell line DNAs with a c-*myc* probe. In addition to the 7.5-kb band, a fainter band of approximately 5 kb (not shown) was identified in the SW480 DNA, probably corresponding to a genomic reorganization. The ethidium bromide staining of the digested genomic DNA before transfer is shown at left. TAP-PCR and Southern blot analyses were performed as described in Material and methods the third exon. The identity of the c-*myc* band was checked by simultaneous electrophoresis of the TAP-PCR product with the product of a conventional high-stringency PCR (annealing temperature, 65°C). The co-migrating band was isolated, cloned and sequenced using standard procedures (Peinado et al, 1992; Perucho et al, 1995).

Differences in the intensity of bands between the tumour and its paired normal tissue were ascertained by direct eye inspection of the film. Relative increases in intensity of the c-*myc* band in the tumour sample compared with its normal sample was considered a symptom of gene amplification compared with the co-amplified arbitrary bands. In addition, in order to have an objective measurement of the magnitude of the change, films were scanned and the intensity of bands quantified using Phoretix 1D software (Newcastle upon Tyne, UK). The intensity of each c-*myc* band was divided by the sum of five arbitrary bands (Figure 2) in order to have a relative measure when the overall intensity of the lanes was not comparable. The ratio between normal tissue and tumour tissue was used to determine the degree of amplification of the c-*myc* gene in each case.

### Method assessment

To test the sensitivity of this technique, duplicated serial dilutions of the SW480 DNA in the DLD-1 DNA were analysed using TAP-PCR and Southern blot hybridization. TAP-PCR was performed as described earlier. For Southern blot analysis, 10 µg of DNA was digested with *Xba*I restriction endonuclease (New England Biolabs, Beverly, MA, USA) and electrophoresed on a 0.7% agarose gel. DNA denaturation and transfer to a blotting membrane (Quiabrane, Quiagen, Santa Clarita, CA, USA) was performed using standard procedures (Sambrook et al, 1989). The filter was hybridized overnight with a c-*myc* probe labelled using the random primer method (Prime-It, Stratagene, La Jolla, CA, USA) with [$\alpha$-$^{32}$P]dCTP (Amersham). The probe was obtained by cloning a PCR product generated with the same primers used for TAP-PCR. The probe was sequenced to confirm its identity.

### Statistical analysis

Statistical analysis was performed using the chi-square test, ANOVA or unpaired *t*-test as appropriate. Contingency tables were analysed using Fisher's exact test or the chi-square test. Fifty-seven cases were included in the follow-up study (Table 1). Given the limited size of our series with complete follow-up and taking into account that five cases presented a late recurrence of the disease (6 or 7 years after surgery), to determine the potential application of c-*myc* amplification as a prognostic factor it was considered more appropriate to use the disease-free survival interval rather than the overall survival. Disease-free survival distributions were calculated using the Kaplan–Meier method and

 *© Cancer Research Campaign 1998*

analysed using the log-rank test. Multivariate analysis was performed using the Cox proportional hazards model. Statistical analysis was performed with SPSS software. All *P*-values are estimated from two-sided statistical tests.

### RESULTS

### Method assessment

Sequencing of the TAP-PCR band co-migrating with the specific PCR c-*myc* product (Figure 1) demonstrated its identity. Technique sensitivity was determined by serial dilutions of the SW480 DNA in the DLD-1 DNA and compared with southern blot hybridization. Parallel results were obtained with both techniques (Figure 2). The maximum dilution of SW480 in DLD-1 that appeared to be distinguishable from uncontaminated DLD-1 was 1:31. As determined by densitometric analysis of the Southern blot hybridization, the c-*myc* gene is about 13.5 ± 0.8 (mean ± s.d.)-fold amplified in SW480 vs DLD-1, whereas using TAP-PCR it was 10.8 ± 0.7. This implies that a 1:31 dilution of SW480 DNA in DLD-1 DNA will correspond to a 1.4 c-*myc* amplification for DLD-1. As shown in Figure 2, TAP-PCR analysis is able to resolve such tiny differences, indicating that the sensitivity of the method is below a 1.5-fold amplification.

### c-myc amplification in colorectal carcinomas

Taking into account the sensitivity of the technique and that our tumour samples were likely to contain a significant proportion of non-tumour cells, a minimal 1.5-fold increase in intensity in the tumour compared with the normal was required to be considered as an amplification. An intensity of the c-*myc* band in the tumour tissue vs its paired normal tissue (> 1.5-fold) was observed in 18 out of 70 cases analysed (26%). An illustrative example is shown in Figure 3. The amplification ratio ranged from two- to fivefold with an average of 3.05 ± 1.02 (mean ± s.d.). Owing to the heterogeneous proportion of tumour cells in a given sample, tumours have been classified as positive or negative for c-*myc* amplification, taking into account that we have a minimal estimation of the amplification ratio. Clinicopathological and molecular associations of the c-*myc* amplification are summarized in Table 2. Lymph node-positive cases showed a higher incidence of c-*myc* amplification (47%) vs no lymph invasion (10%) ($P = 0.0005$). No differences were observed between Dukes' stages C and D. In tumours containing a mutation in the *p53* gene, c-*myc* was more often amplified ($P = 0.0484$). No significant correlation was found with other variables such as age, sex, race, tumour differentiation and location and presence of mutations in the *ras* gene. None of the eight tumours displaying microsatellite instability contained an amplified c-*myc*; nevertheless, the differences did not reach statistical significance.

Univariate survival analysis for the different parameters studied (Table 3) indicated that advanced Dukes' stages and poor differentiation were markers of bad prognosis. Although cases with amplification of the c-*myc* gene also had a poorer disease-free survival (log-rank, $P = 0.0628$), due to its strong association with more advanced stages, it does not constitute an independent prognostic factor as determined by multivariate Cox analysis (Table 3).

**Figure 3** Duplicated TAP-PCR analysis of three normal tissue (N) and tumour tissue (T) pairs. Numbers at top indicate case. Case 125 displays an increase in the c-*myc* band (marked with an arrow) that after densitometric analysis revealed a five-fold amplification in the tumour sample vs its paired normal mucosa DNA. SW480 and DLD-1 cell lines were used as controls

**Table 2** C-*myc* amplification in colorectal carcinomas

| Parameter | c-*myc* amplification[a] | Significance *P* |
|---|---|---|
| Race | | |
| Black | 3/16 (19) | 0.5050 |
| White | 13/48 (19) | |
| Sex | | |
| Female | 6/20 (30) | 0.6038 |
| Male | 12/50 (24) | |
| Dukes' stage | | |
| A–B | 4/40 (10) | 0.0024 |
| C | 9/19 (47) | |
| D | 5/11 (45) | |
| Lymph node invasion | | |
| Negative | 4/40 (10) | 0.0005 |
| Positive | 14/30 (47) | |
| Location | | |
| Left | 12/38 (31) | 0.1220 |
| Right | 4/27 (15) | |
| Differentiation | | |
| Well–Moderate | 12/51 (23) | 0.6639 |
| Poor | 3/10 (30) | |
| *ras* mutation | | |
| Negative | 14/46 (30) | 0.2109 |
| Positive | 4/24 (17) | |
| *p53* mutation | | |
| Negative | 3/24 (12) | 0.0484 |
| Positive | 14/40 (35) | |
| MMP[b] | | |
| Negative | 18/62 (29) | 0.0770 |
| Positive | 0/8 (0) | |

[a]Numbers indicate cases displaying c-*myc* amplification respect the total for each category. Numbers in parentheses indicate percentages. [b]Microsatellite mutator phenotype.

© Cancer Research Campaign 1998

**Table 3** Disease-free survival: univariate and multivariate analysis for clinical pathological and genetic parameters

| Prognostic factor | Univariate analysis: Number of observations | 5-year survival (percentage) | Log-rank *P* |
|---|---|---|---|
| Dukes' stage (Astler–Coller) | | | |
| A–B | 35 | 71.1 | <0.0001 |
| C | 15 | 51.8 | |
| D | 7 | 0 | |
| Degree of differentiation | | | |
| Well–moderate | 43 | 59.2 | 0.0344 |
| Poor | 7 | 28.6 | |
| Location | | | |
| Left | 29 | 64.1 | 0.5137 |
| Right | 24 | 58.3 | |
| *ras* mutation | | | |
| Negative | 38 | 62.4 | 0.7471 |
| Positive | 19 | 47.4 | |
| *p53* mutation | | | |
| Negative | 19 | 61.7 | 0.7994 |
| Positive | 32 | 56.2 | |
| MMP | | | |
| Negative | 50 | 57.0 | 0.9765 |
| Positive | 7 | 57.1 | |
| c-*myc* amplification | | | |
| Negative | 42 | 61.1 | 0.0628 |
| Positive | 15 | 45.7 | |

| Step | Variable | Hazard ratio | Cox multivariate analysis: 95% CI | *P* |
|---|---|---|---|---|
| 1 | Dukes' stage (Astler-Coller) | | | |
| | A–B | | | |
| | C | 1.8 | 0.7–4.3 | 0.1987 |
| | D | 6.7 | 2.4–18.7 | 0.0003 |
| 2 | c-*myc* amplification | | | |
| | Negative | | | |
| | Positive | 1.2 | 0.5–2.8 | 0.6315 |

Overall survival analysis produced similar results (univariate log-rank analysis, $P = 0.0673$; multivariate Cox analysis, hazard ratio = 1.2, $P = 0.7459$).

### DISCUSSION

Here, we report a variation of the AP-PCR fingerprinting technique by introducing a bias in the design of the primers towards the amplification of a particular sequence. This variation makes this technique suitable for the allelic dosage of specific genomic sequences. A similar approach has been used to isolate cDNA members of a gene family (Stone et al, 1994) and to quantify collagenase expression in tumour cells (Vinyals et al, submitted). The quality of the technique has been assessed by performing reproducibility and sensitivity tests. Finally, it has been validated by comparison with Southern blot hybridization analyses. The c-*myc* copy number in the SW480 cell line was of the same order when measured using Southern and TAP-PCR and in the same range as previously described (Untawale and Blick, 1988). It can be concluded that TAP-PCR is a reliable and sensitive method for the allelic dosage of c-*myc*. As the pattern is very reproducible from individual to individual, concomitant analysis of the paired normal mucosa is not absolutely necessary.

Nevertheless, in order to discard a polymorphic differential PCR amplification of one or many of the bands, it is highly recommended to use normal tissue from the same patient as the control. Owing to the intrinsic instability of the cancer cell genome, use of multiple and diverse reference loci is an indispensable condition. In consequence, one of the properties of AP-PCR, the random chromosomal origin of the co-amplified bands (Peinado et al, 1992; Yasuda et al, 1996), is of special relevance to guarantee the accuracy of the quantification.

The incidence of c-*myc* amplification in our series of colorectal tumours is in the upper range (about 25%) of the data reported in the literature, in agreement with Heerdt et al (1991) and Wang et al (1994). As the amplifications we have seen are moderate (threefold in average), a lack of sufficient sensitivity may explain the failure of other studies to detect such gains. Although not significant, we have observed a lower incidence of c-*myc* amplification in the right-side colon (Table 2). This is in agreement with a previous study in which c-*myc* overexpression was more frequent in the left colon (Rothberg et al, 1985). In fact, these authors have already defined two pathways for sporadic colorectal tumours in relationship to the familial syndromes. Tumours behaving like hereditary non-polyposis colorectal cancer (HNPCC) were located at the right side and did not overexpress c-*myc*, whereas tumours behaving like familial polyposis coli were left sided and showed c-*myc* activation. In agreement with this observation, more recent evidence has shown that a significant proportion of the right-side-located tumours progress through a different molecular pathway characterized by a DNA mismatch repair-deficient machinery (Aaltonen et al, 1993; Ionov et al, 1993; Lothe et al, 1993; Peltomaki et al, 1993; Thibodeau et al, 1993; Perucho et al, 1994). These tumours do not show c-*myc* amplification (Table 2) and, in consequence, might be responsible for this trend.

Although there is a general agreement that c-*myc* amplification is more frequent in more aggressive tumours, including colorectal, gastric and lung (Heerdt et al, 1991; Yokota et al, 1986; Little et al, 1983; Shibuya et al, 1985), because of sample and technical heterogeneity data are barely comparable. Tumours depicting c-*myc* amplification show a poorer disease-free and overall survival. Unfortunately, the relevance of this association has a doubtful application. The clear correlation with advanced Dukes' stages indicates the dependency between both variables. This observation also holds for overall survival. In consequence, assessment of c-*myc* dosage status does not seem to be useful as a prognostic factor.

c-*myc* amplification is one of the multiple alterations that accumulates during the tumour progression and its late appearance suggests that it does not play a significant role in the transformation of the cell. Nevertheless, experimental evidence shows that c-*myc* activation (which is not necessarily produced by gene amplification) has a direct and important implication in multistage carcinogenesis (Field and Spandidos, 1990). The apparent irrelevance, at clinical level, of c-*myc* activation in colorectal cancer is in a way expected and explained, at least in part, by its late occurrence. It can be hypothesized that either the activation mechanism (amplification) is a late event, or the selective advantage that it confers to the tumour cell is only displayed in late stages. This is based on studies that indicate that c-*myc* activation induces apoptosis (Evan et al, 1992). In consequence, for tumour cells,

© Cancer Research Campaign 1998

apoptosis overriding by activation/inactivation of other factors (see Stewart, 1994 for review) would be a prerequisite before c-*myc* amplification can occur. In fact, this hypothesis is supported by our observation that c-*myc* amplification is associated with mutations in the *p53* gene (one of the inductors of apoptosis). In this case, p53 inactivation [that presumably occurs in the adenoma–carcinoma transition (Fearon and Vogelstein, 1990)] will play a double role to facilitate c-*myc* activation, on one side by inducing apoptosis abrogation (Yonish-Rouach et al, 1991; Shaw et al, 1992) and on the other by overriding of the genomic stability controls (Lane, 1992) that might prevent gene amplification (Livingstone et al, 1992; Yin et al, 1992). The positive correlation between c-*myc* activation and mutations in the *p53* gene (Table 2) supports this hypothesis.

Although we have stated gene 'amplification' as all copy number increases observed at the c-*myc* level, this should be regarded with caution, especially in view of the modest nature of such increases. In addition, random screening of the cancer cell genome by AP-PCR has revealed that many anonymous bands located in chromosome 8 (as it is the c-*myc* gene) also display frequent gains, which in some cases are associated with c-*myc* amplification (two out of five) (unpublished data). This implies that the 'amplified' fragment may include a wide chromosomal region. If this is the case, such gains might affect a large region, if not all, of chromosome 8. This argument has relevant implications for two reasons: first, the number of genes displaying gains would be very high, and, in addition to c-*myc*, many of these genes may affect tumour behaviour. Second, the mechanism causing such imbalances is probably different from the one producing small regional amplifications. Precise characterization of the involved chromosomal fragment should answer such questions. Interestingly, extensive AP-PCR genome analysis of human cell lines carrying amplified c-*myc* genes showed that all of them displayed concurrent amplification of other DNA fragments mapped to chromosome 8 (Okazaki et al, 1996). Okazaki et al (1996) hypothesize that these sequences are part of an amplification unit that includes the c-*myc* gene. The total size of the amplified region in a small-cell lung carcinoma cell line was estimated to be 7.5 Mb.

A possible inaccuracy in our results is the quantification of the c-*myc* copy number in tumour samples. Heterogeneous population and contamination by normal cells might mask the real degree of amplification. For this reason, we have only classified tumours as positive or negative for c-*myc* amplification, taking into account that we have a minimal estimation of the amplification ratio. Although the technique we have used is sensitive enough to detect a relatively small amplification in a relatively small proportion of the cells, it is obvious that this limitation will affect in a lower grade more advanced tumours and consequently may mislead in the interpretation of the results.

In summary, TAP-PCR appears to be an appropriate technique to determine gene amplification. The low requirement of DNA template and the simplicity of the technique indicates that it may be especially useful in the analysis of small pieces that provide insufficient amounts of material for analysis using other techniques. Our study clearly demonstrates a significant incidence of c-*myc* amplification in colorectal cancer and its association with invasiveness. Owing to the complexity of c-*myc* function in cellular processes, further studies are required to elucidate the participation of c-*myc* amplification in aetiogenesis and the consequences that it may have for tumour progression.

### ACKNOWLEDGEMENTS

We thank Gabriel Capellà for encouragement and critical review of the manuscript. This work was supported by grants from FIS (94/37), CICYT (SAF 96/187) and Marató de TV3 1994 (to MAP) and NIH grants CA38579 and CA63585 from the National Cancer Institute (to MP). RA and ST are fellows of the Spanish Ministry of Education.

### REFERENCES

Aaltonen LA, Peltomaki P, Leach FS, Sistonen P, Pylkkanen L, Mecklin JP, Jarvinen H, Powell SM, Jen J, Hamilton SR, Petersen GM, Kinzler KW, Vogelstein B and De La Chapelle A (1993) Clues to the pathogenesis of familial colorectal cancer. *Science* **260**: 812–816

Achille A, Biasi MO, Zamboni G, Bogina G, Magalini AR, Pederzoli P, Perucho M and Scarpa A (1996) Chromosome 7q allelic losses in pancreatic carcinoma. *Cancer Res* **56**: 3808–3813

Alitalo K, Schwab M, Lin CC, Varmus HE and Bishop JM (1983) Homogeneously staining chromosomal regions contain amplified copies of an abundantly expressed cellular oncogene (c-myc) in malignant neuroendocrine cells from a human colon carcinoma. *Proc Natl Acad Sci USA* **80**: 1707–1711

Arribas R, Capella G, Tortola S, Masramon L, Grizzle WE, Perucho M and Peinado MA (1997) Assessment of genomic damage in colorectal cancers by DNA fingerprinting: prognostic applications. *J Clin Oncol* **15**: 3230–3240

Basik M, Stoler DL, Kontzoglou KC, Rodriguez-Bigas MA, Petrelli NJ and Anderson GR (1997) Genomic instability in sporadic colorectal cancer quantitated by inter-simple sequence repeat PCR analysis. *Genes Chromos Cancer* **18**: 19–29

Bishop JM (1991) Molecular themes in oncogenesis. *Cell* **64**: 235–248

Bocker T, Schlegel J, Kullmann F, Stumm G, Zirngibl H, Epplen JT and Ruschoff J (1996) Genomic instability in colorectal carcinomas: comparison of different evaluation methods and their biological significance. *J Pathol* **179**: 15–19

Capella G, Cronauer-Mitra S, Peinado MA and Perucho M (1991) Frequency and spectrum of mutations at codons 12 and 13 of the c-k-ras gene in human tumors. *Environ Health Perspect* **93**: 125–131

Collins S and Groudine M (1982) Amplification of endogenous myc-related DNA sequences in a human myeloid leukaemia cell line. *Nature* **298**: 679–681

Erisman MD, Rothberg PG, Diehl RE, Morse CC, Spandorfer JM and Astrin SM (1985) Deregulation of c-myc gene expression in human colon carcinoma is not accompanied by amplification or rearrangement of the gene. *Mol Cell Biol* **5**: 1969–1976

Erisman MD, Litwin S, Keidan RD, Comis RL and Astrin SM (1988) Noncorrelation of the expression of the c-myc oncogene in colorectal carcinoma with recurrence of disease or patient survival. *Cancer Res* **48**: 1350–1355

Evan GI, Wyllie AH, Gilbert CS, Littlewood TD, Land H, Brooks M, Waters CM, Penn LZ and Hancock DC (1992) Induction of apoptosis in fibroblasts by c-myc protein. *Cell* **69**: 119–128

Fearon ER and Vogelstein B (1990) A genetic model for colorectal tumorigenesis. *Cell* **61**: 759–767

Ferré F, Marchese A, Pezzoli P, Griffin S, Buxton E and Boyer V (1994) Quantitative PCR: an overview. in *The Polymerase Chain Reaction*, Mullis KB, Ferré F and Gibbs RA (ed), pp. 67–88. Birkhauser: Boston

Field JK and Spandidos DA (1990) The role of ras and myc oncogenes in human solid tumours and their relevance in diagnosis and prognosis. *Anticancer Res* **10**: 1–22

Finley GG, Schulz NT, Hill SA, Geiser JR, Pipas JM and Meisler AI (1989) Expression of the myc gene family in different stages of human colorectal cancer. *Oncogene* **4**: 963–971

Garte SJ (1993) The c-myc oncogene in tumor progression. *Crit Rev Oncog* **4**: 435–449

Heerdt BG, Molinas S, Deitch D and Augenlicht LH (1991) Aggressive subtypes of human colorectal tumors frequently exhibit amplification of the c-myc gene. *Oncogene* **6**: 125–129

Ionov Y, Peinado MA, Malkhosyan S, Shibata D and Perucho M (1993) Ubiquitous somatic mutations in simple repeated sequences reveal a new mechanism for colonic carcinogenesis. *Nature* **363**: 558–561

Kohno T, Morishita K, Takano H, Shapiro DN and Yokota J (1994) Homozygous deletion at chromosome 2q33 in human small-cell lung carcinoma identified by arbitrarily primed PCR genomic fingerprinting. *Oncogene* **9**: 103–108

© Cancer Research Campaign 1998

Kozma L, Kiss I, Szakall S and Ember I (1994) Investigation of c-myc oncogene amplification in colorectal cancer. *Cancer Lett* **81**: 165–169

Lane DP (1992) p53, guardian of the genome. *Nature* **358**: 15–16

Little CD, Nau MM, Carney DN, Gazdar AF and Minna JD (1983) Amplification and expression of the c-myc oncogene in human lung cancer cell lines. *Nature* **306**: 194–196

Livingstone LR, White A, Sprouse J, Livanos E, Jacks T and Tlsty TD (1992) Altered cell cycle arrest and gene amplification potential accompany loss of wild-type p53. *Cell* **70**: 923–935

Lothe RA, Peltomaki P, Meling GI, Aaltonen LA, Nystrom-Lahti M, Pylkkanen L, Heimdal K, Andersen TI, Moller P, Rognum TO, Fossa SD, Haldorsen T, Langmark F, Brogger A, De La Chapelle A and Borrensen AL (1993) Genomic instability in colorectal cancer: relationship to clinicopathological variables and family history. *Cancer Res* **53**: 5849–5852

Matsumura T, Dohi K, Takanashi A, Ito H and Tahara E (1990) Alteration and enhanced expression of the c-myc oncogene in human colorectal carcinomas. *Pathol Res Pract* **186**: 205–211

Nagai MA, Habr-Gama A, Oshima CT and Brentani MM (1992) Association of genetic alterations of c-myc, c-fos, and c-Ha-ras proto-oncogenes in colorectal tumors. Frequency and clinical significance. *Dis Colon Rectum* **35**: 444–451

Nakano H, Yamamoto F, Neville C, Evans D, Mizuno T and Perucho M (1984) Isolation of transforming sequences of two human lung carcinomas: structural and functional analysis of the activated c-K-ras oncogenes. *Proc Natl Acad Sci USA* **81**: 71–75

Okazaki T, Takita J, Kohno T, Handa H and Yokota J (1996) Detection of amplified genomic sequences in human small-cell lung carcinoma cells by arbitrarily primed-PCR genomic fingerprinting. *Hum Genet* **98**: 253–258

Peinado MA, Malkhosyan S, Velazquez A and Perucho M (1992) Isolation and characterization of allelic losses and gains in colorectal tumors by arbitrarily primed polymerase chain reaction. *Proc Natl Acad Sci USA* **89**: 10065–10069

Peinado MA, Fernandez-Renart M, Capella G, Wilson L and Perucho M (1993) Mutations in the p53 suppressor gene do not correlate with c-K-ras mutations in colorectal cancer. *Int J Oncol* **2**: 123–134

Peltomaki P, Lothe RA, Aaltonen LA, Pylkkanen L, Nystrom-Lahti M, Seruca R, David L, Holm R, Ryberg D, Haugen A, Brogger A, Borresen AL and De La Chapelle A (1993) Microsatellite instability is associated with tumors that characterize the hereditary non-polyposis colorectal carcinoma syndrome. *Cancer Res* **53**: 5853–5858

Perucho M, Peinado MA, Ionov Y, Casares S, Malkhosyan S and Stanbridge E (1994) Defects in replication fidelity of simple repeated sequences reveal a new mutator mechanism for oncogenesis. *Cold Spring Harb Symp Quant Biol* **59**: 339–348

Perucho M, Welsh J, Peinado MA, Ionov Y and McClelland M (1995) Fingerprinting of DNA and RNA by arbitrarily primed polymerase chain reaction: applications in cancer research. *Methods Enzymol* **254**: 275–290

Rhoer-Moja S, Cohen-Haguenauer O, Jouve C, Healy JC and Vindimian M (1993) Detection of quantitative polymerase chain reaction products by hybridization on magnetic support with 125I-radiolabeled probes: quantification of c-myc copy numbers. *Anal Biochem* **213**: 12–18

Rothberg G, Spandorfer JM, Erisman MD, Staroscik RN, Sears HF, Petersen RO and Astrin SM (1985) Evidence that c-myc expression defines two genetically distinct forms of colorectal adenocarcinoma. *Br J Cancer* **52**: 629–632

Sambrook J, Fritsch EF and Maniatis T (1989) *Molecular Cloning. A Laboratory Manual*. Cold Spring Harbor Laboratory Press: Cold Spring Harbor, NY

Sato K, Miyahara M, Saito T and Kobayashi M (1994) c-myc mRNA overexpression is associated with lymph node metastasis in colorectal cancer. *Eur J Cancer* **30**: 1113–1117

Sestini R, Orlando C, Zentilin L, Lami D, Gelmini S, Pinzani P, Giacca M and Pazzagli M (1995) Gene amplification for c-erbB-2, c-myc, epidermal growth factor receptor, int-2, and N-myc measured by quantitative PCR with a multiple competitor template. *Clin Chem* **41**: 826–832

Shaw P, Bovey R, Tardy S, Sahli R, Sordat B and Costa J (1992) Induction of apoptosis by wild-type p53 in a human colon tumor-derived cell line. *Proc Natl Acad Sci USA* **89**: 4495–4499

Shibata D, Peinado MA, Ionov Y, Malkhosyan S and Perucho M (1994) Genomic instability in repeated sequences is an early somatic event in colorectal tumorigenesis that persists after transformation. *Nature Genet* **6**: 273–281

Shibuya M, Yokota J and Ueyama Y (1985). Amplification and expression of a cellular oncogene (c-myc) in human gastric adenocarcinoma cells. *Mol Cell Biol* **5**: 414–418

Smith DR, Myint T and Goh HS (1993) Over-expression of the c-myc proto-oncogene in colorectal carcinoma. *Br J Cancer* **68**: 407–413

Stewart BW (1994) Mechanisms of apoptosis: integration of genetic, biochemical, and cellular indicators. *J Natl Cancer Inst* **86**: 1286–1296

Stone B and Wharton W (1994) Targeted RNA fingerprinting: the cloning of differentially expressed cDNA fragments enriched for members of the zinc finger gene family. *Nucleic Acids Res* **22**: 2612–2618

Sugimoto T, Tsukamato F, Fujita M and Takai S (1994) Ki-ras and c-myc oncogene expression measured by coamplification polymerase chain reaction. *Biochem Biophys Res Commun* **201**: 574–580

Thibodeau SN, Bren G and Schaid D (1993) Microsatellite instability in cancer of the proximal colon. *Science* **260**: 816–819

Untawale S and Blick M (1988) Oncogene expression in adenocarcinomas of the colon and in colon tumor-derived cell lines. *Anticancer Res* **8**: 1–7

Wang J, Li L, Li S, Cui H and Shen G (1994) A study of c-myc oncogene expression and amplification in colorectal cancer. *Chin Med Sci J* **9**: 24–28

Watson PH, Safneck JR, Le K, Dubik D and Shiu RP (1993) Relationship of c-myc amplification to progression of breast cancer from in situ to invasive tumor and lymph node metastasis. *J Natl Cancer Inst* **85**: 902–907

Welsh J and McClelland M (1990) Fingerprinting genomes using PCR with arbitrary primers. *Nucleic Acids Res* **18**: 7213–7218

Yasuda J, Navarro JM, Malkhosyan S, Velazquez A, Arribas R, Sekiya T and Perucho M (1996) Chromosomal assignment of human DNA fingerprint sequences by simultaneous hybridization to arbitrarily primed PCR products from human/rodent monochromosome cell hybrids. *Genomics* **34**: 1–8

Yin Y, Tainsky MA, Bischoff FZ, Strong LC and Wahl GM (1992) Wild-type p53 restores cell cycle control and inhibits gene amplification in cells with mutant p53 alleles. *Cell* **70**: 937–948

Yokota J, Tsunetsugu-Yokota Y, Battifora H, Le Fevre C and Cline MJ (1986) Alterations of myc, myb, and rasHa proto-oncogenes in cancers are frequent and show clinical correlation. *Science* **231**: 261–265

Yonish-Rouach E, Resnitzky D, Lotem J, Sachs L, Kimchi A and Oren M (1991) Wild-type p53 induces apoptosis of myeloid leukaemic cells that is inhibited by interleukin-6. *Nature* **352**: 345–347

 *© Cancer Research Campaign 1998*


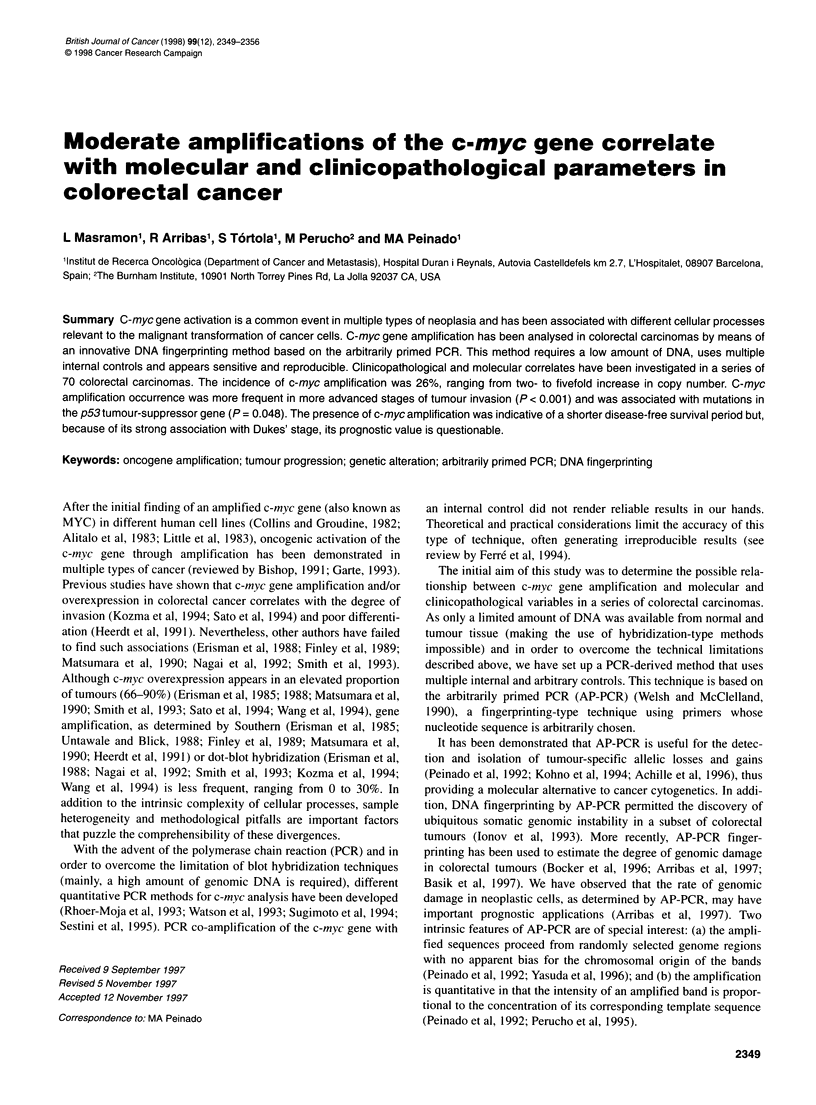

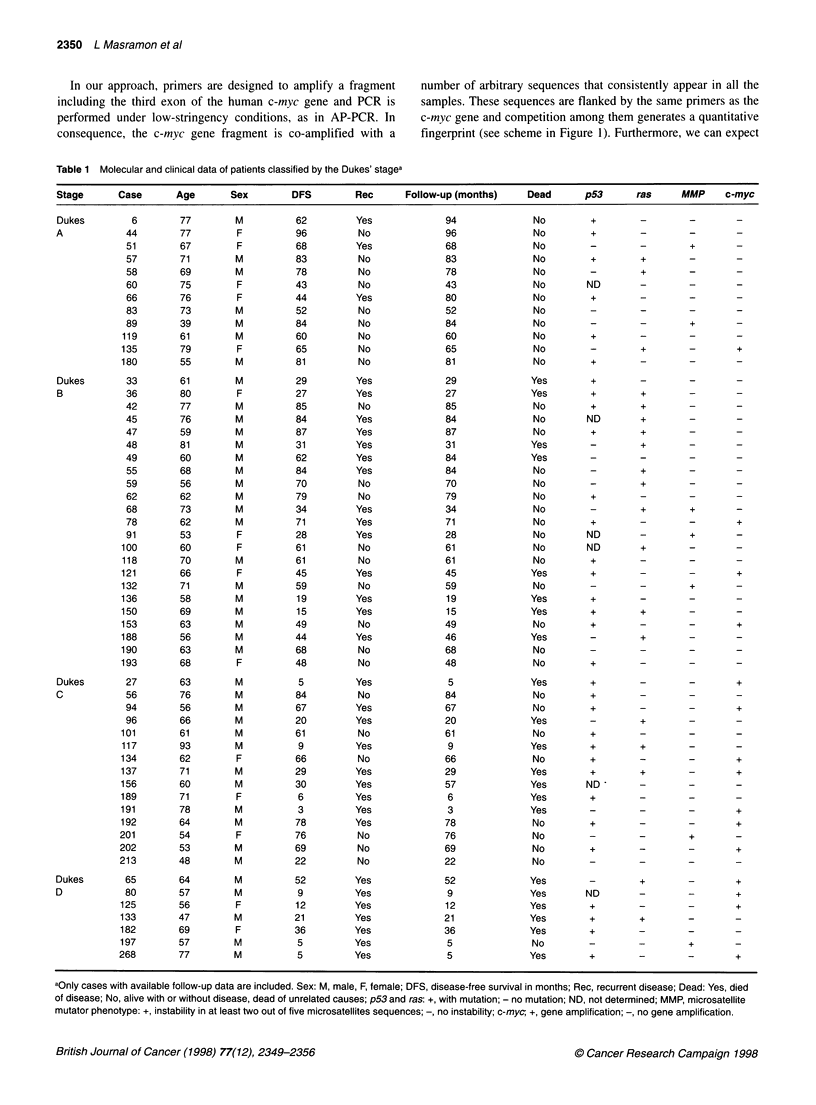

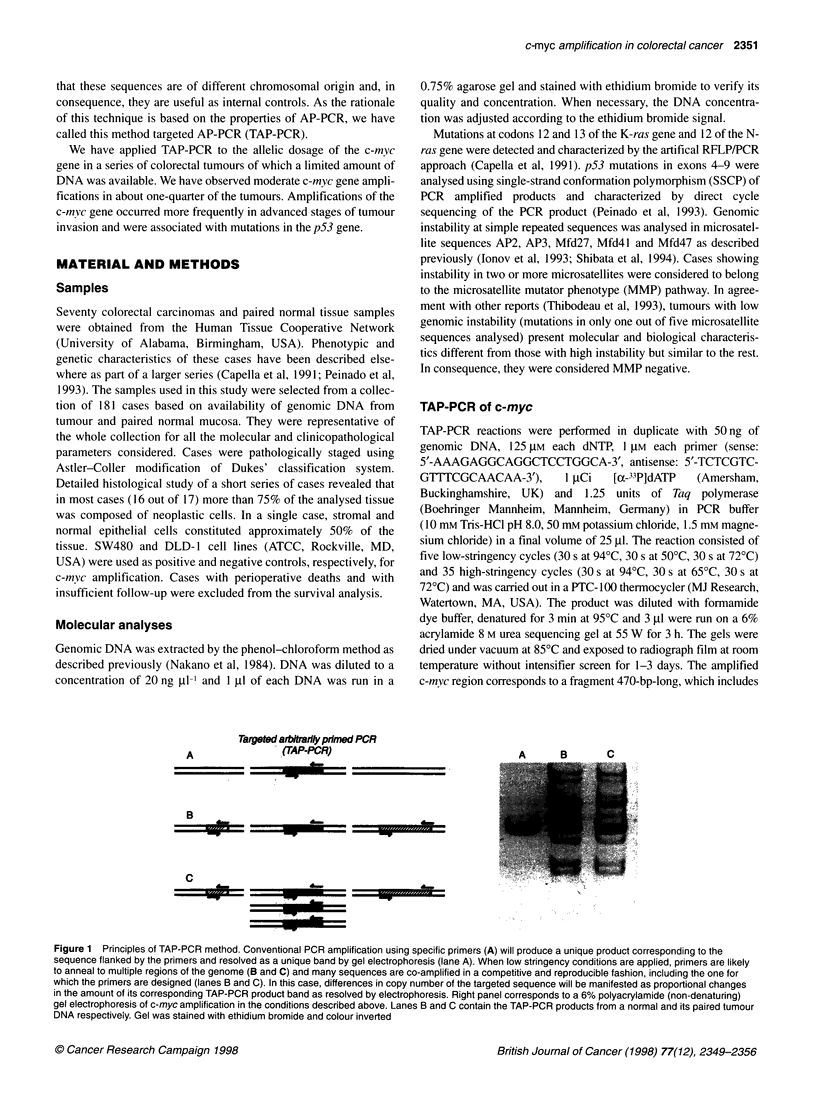

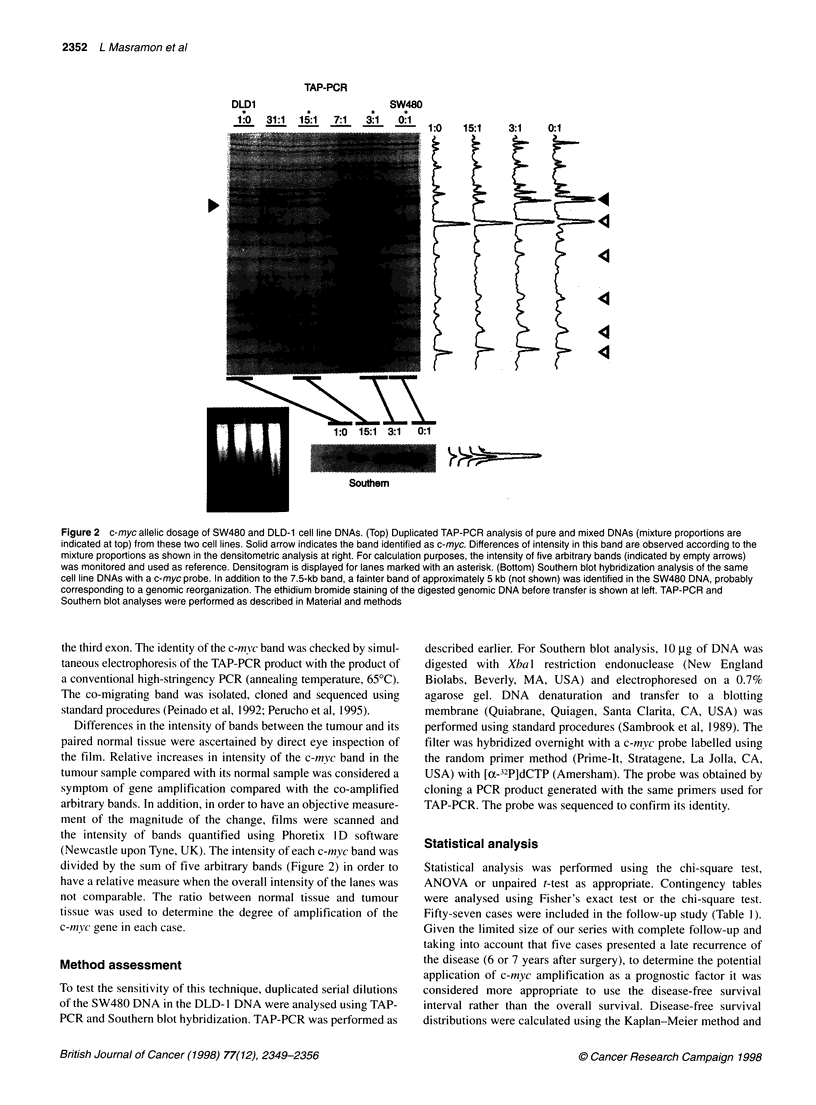

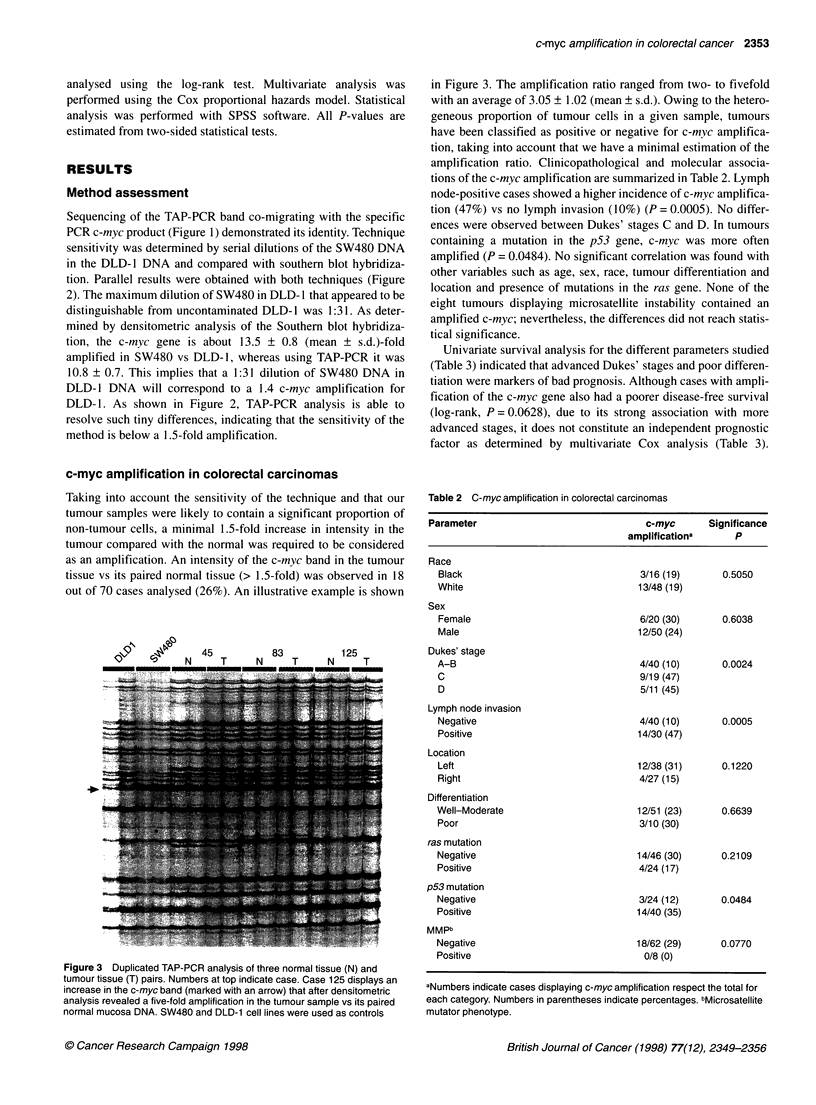

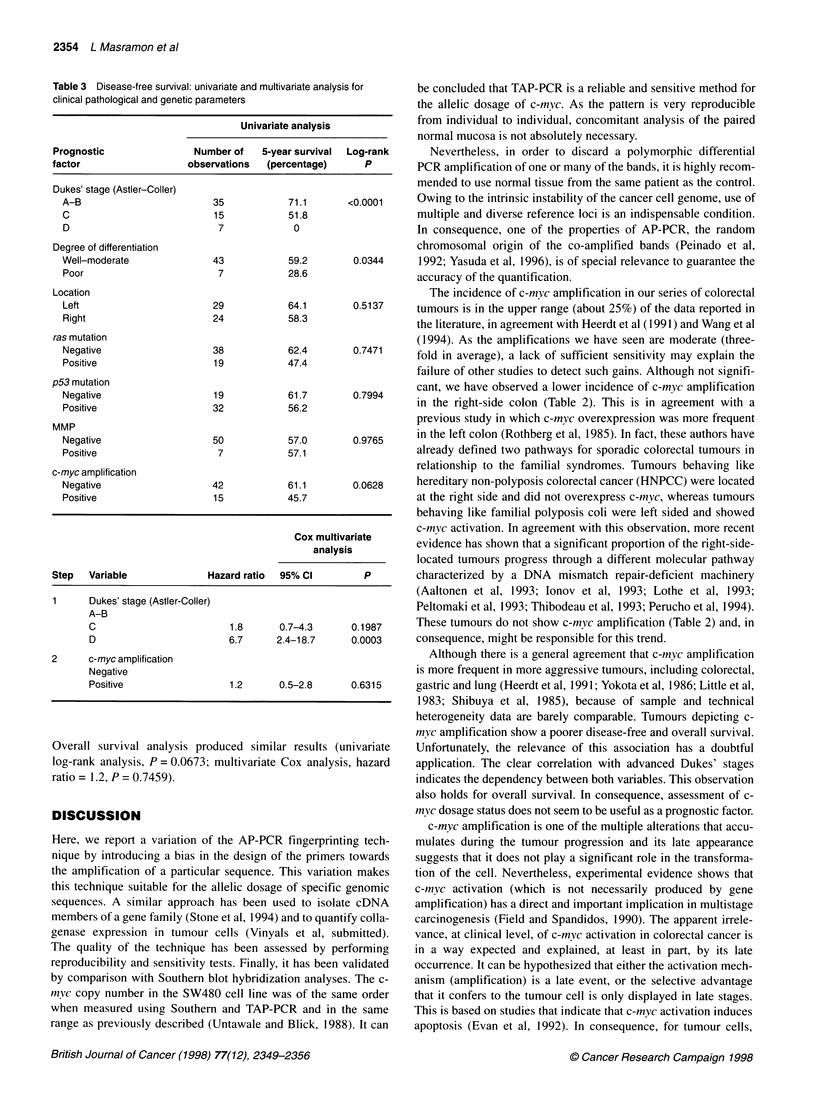

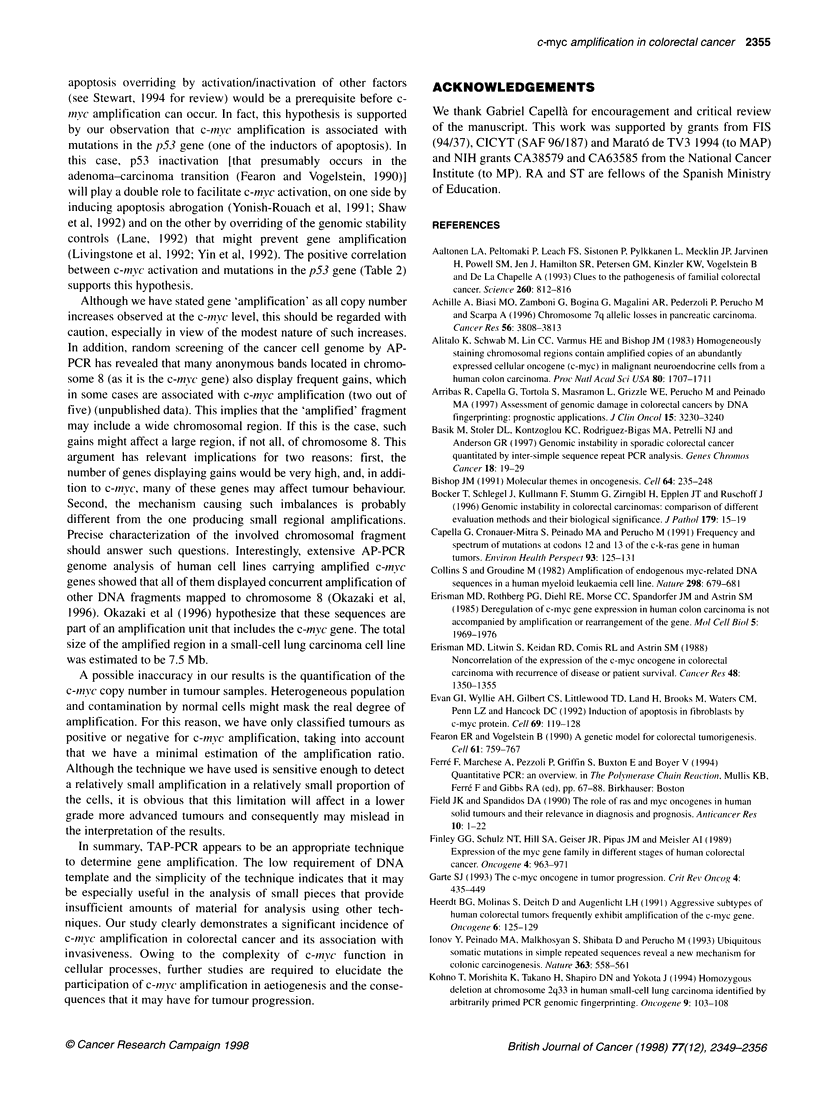

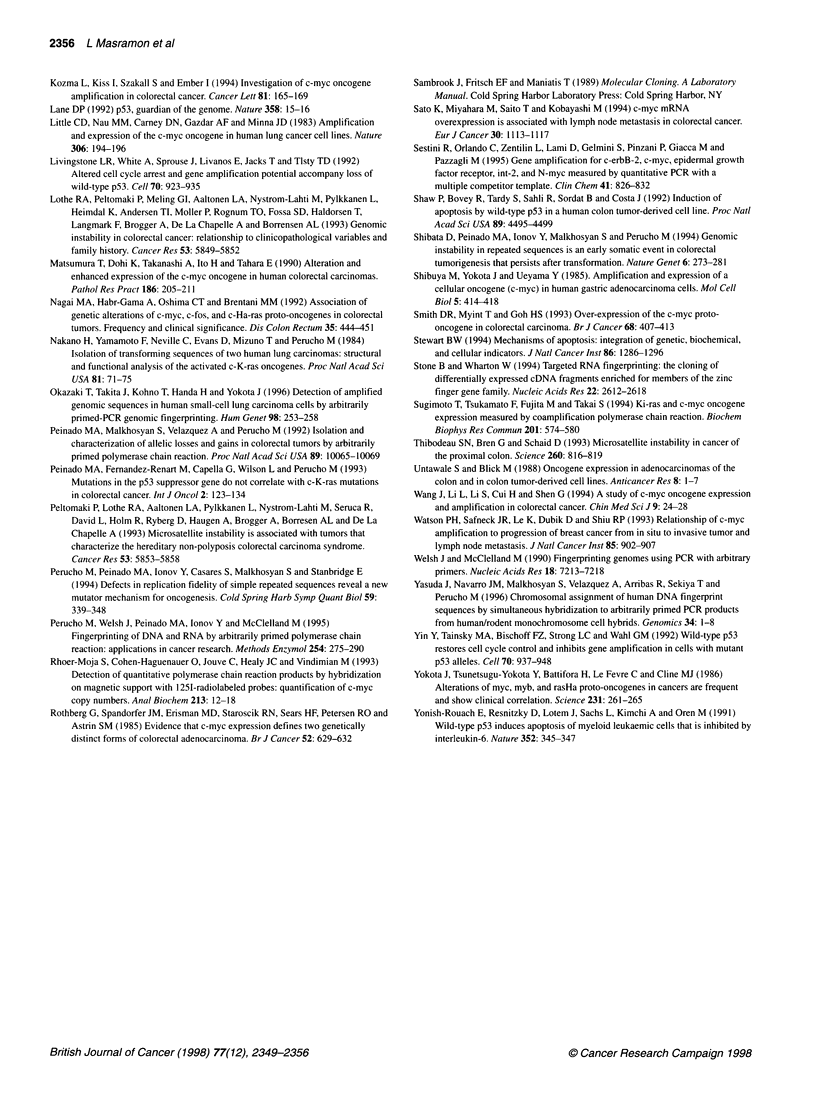


## References

[OCR_01225] Aaltonen L. A., Peltomäki P., Leach F. S., Sistonen P., Pylkkänen L., Mecklin J. P., Järvinen H., Powell S. M., Jen J., Hamilton S. R. (1993). Clues to the pathogenesis of familial colorectal cancer.. Science.

[OCR_01232] Achille A., Biasi M. O., Zamboni G., Bogina G., Magalini A. R., Pederzoli P., Perucho M., Scarpa A. (1996). Chromosome 7q allelic losses in pancreatic carcinoma.. Cancer Res.

[OCR_01234] Alitalo K., Schwab M., Lin C. C., Varmus H. E., Bishop J. M. (1983). Homogeneously staining chromosomal regions contain amplified copies of an abundantly expressed cellular oncogene (c-myc) in malignant neuroendocrine cells from a human colon carcinoma.. Proc Natl Acad Sci U S A.

[OCR_01241] Arribas R., Capellà G., Tórtola S., Masramon L., Grizzle W. E., Perucho M., Peinado M. A. (1997). Assessment of genomic damage in colorectal cancer by DNA fingerprinting: prognostic applications.. J Clin Oncol.

[OCR_01246] Basik M., Stoler D. L., Kontzoglou K. C., Rodriguez-Bigas M. A., Petrelli N. J., Anderson G. R. (1997). Genomic instability in sporadic colorectal cancer quantitated by inter-simple sequence repeat PCR analysis.. Genes Chromosomes Cancer.

[OCR_01253] Bishop J. M. (1991). Molecular themes in oncogenesis.. Cell.

[OCR_01255] Bocker T., Schlegel J., Kullmann F., Stumm G., Zirngibl H., Epplen J. T., Rüschoff J. (1996). Genomic instability in colorectal carcinomas: comparison of different evaluation methods and their biological significance.. J Pathol.

[OCR_01260] Capella G., Cronauer-Mitra S., Pienado M. A., Perucho M. (1991). Frequency and spectrum of mutations at codons 12 and 13 of the c-K-ras gene in human tumors.. Environ Health Perspect.

[OCR_01265] Collins S., Groudine M. (1982). Amplification of endogenous myc-related DNA sequences in a human myeloid leukaemia cell line.. Nature.

[OCR_01275] Erisman M. D., Litwin S., Keidan R. D., Comis R. L., Astrin S. M. (1988). Noncorrelation of the expression of the c-myc oncogene in colorectal carcinoma with recurrence of disease or patient survival.. Cancer Res.

[OCR_01269] Erisman M. D., Rothberg P. G., Diehl R. E., Morse C. C., Spandorfer J. M., Astrin S. M. (1985). Deregulation of c-myc gene expression in human colon carcinoma is not accompanied by amplification or rearrangement of the gene.. Mol Cell Biol.

[OCR_01284] Evan G. I., Wyllie A. H., Gilbert C. S., Littlewood T. D., Land H., Brooks M., Waters C. M., Penn L. Z., Hancock D. C. (1992). Induction of apoptosis in fibroblasts by c-myc protein.. Cell.

[OCR_01287] Fearon E. R., Vogelstein B. (1990). A genetic model for colorectal tumorigenesis.. Cell.

[OCR_01296] Field J. K., Spandidos D. A. (1990). The role of ras and myc oncogenes in human solid tumours and their relevance in diagnosis and prognosis (review).. Anticancer Res.

[OCR_01301] Finley G. G., Schulz N. T., Hill S. A., Geiser J. R., Pipas J. M., Meisler A. I. (1989). Expression of the myc gene family in different stages of human colorectal cancer.. Oncogene.

[OCR_01306] Garte S. J. (1993). The c-myc oncogene in tumor progression.. Crit Rev Oncog.

[OCR_01310] Heerdt B. G., Molinas S., Deitch D., Augenlicht L. H. (1991). Aggressive subtypes of human colorectal tumors frequently exhibit amplification of the c-myc gene.. Oncogene.

[OCR_01315] Ionov Y., Peinado M. A., Malkhosyan S., Shibata D., Perucho M. (1993). Ubiquitous somatic mutations in simple repeated sequences reveal a new mechanism for colonic carcinogenesis.. Nature.

[OCR_01320] Kohno T., Morishita K., Takano H., Shapiro D. N., Yokota J. (1994). Homozygous deletion at chromosome 2q33 in human small-cell lung carcinoma identified by arbitrarily primed PCR genomic fingerprinting.. Oncogene.

[OCR_01329] Kozma L., Kiss I., Szakáll S., Ember I. (1994). Investigation of c-myc oncogene amplification in colorectal cancer.. Cancer Lett.

[OCR_01332] Lane D. P. (1992). Cancer. p53, guardian of the genome.. Nature.

[OCR_01334] Little C. D., Nau M. M., Carney D. N., Gazdar A. F., Minna J. D. (1983). Amplification and expression of the c-myc oncogene in human lung cancer cell lines.. Nature.

[OCR_01339] Livingstone L. R., White A., Sprouse J., Livanos E., Jacks T., Tlsty T. D. (1992). Altered cell cycle arrest and gene amplification potential accompany loss of wild-type p53.. Cell.

[OCR_01344] Lothe R. A., Peltomäki P., Meling G. I., Aaltonen L. A., Nyström-Lahti M., Pylkkänen L., Heimdal K., Andersen T. I., Møller P., Rognum T. O. (1993). Genomic instability in colorectal cancer: relationship to clinicopathological variables and family history.. Cancer Res.

[OCR_01352] Matsumura T., Dohi K., Takanashi A., Ito H., Tahara E. (1990). Alteration and enhanced expression of the c-myc oncogene in human colorectal carcinomas.. Pathol Res Pract.

[OCR_01357] Nagai M. A., Habr-Gama A., Oshima C. T., Brentani M. M. (1992). Association of genetic alterations of c-myc, c-fos, and c-Ha-ras proto-oncogenes in colorectal tumors. Frequency and clinical significance.. Dis Colon Rectum.

[OCR_01361] Nakano H., Yamamoto F., Neville C., Evans D., Mizuno T., Perucho M. (1984). Isolation of transforming sequences of two human lung carcinomas: structural and functional analysis of the activated c-K-ras oncogenes.. Proc Natl Acad Sci U S A.

[OCR_01368] Okazaki T., Takita J., Kohno T., Handa H., Yokota J. (1996). Detection of amplified genomic sequences in human small-cell lung carcinoma cells by arbitrarily primed-PCR genomic fingerprinting.. Hum Genet.

[OCR_01373] Peinado M. A., Malkhosyan S., Velazquez A., Perucho M. (1992). Isolation and characterization of allelic losses and gains in colorectal tumors by arbitrarily primed polymerase chain reaction.. Proc Natl Acad Sci U S A.

[OCR_01383] Peltomäki P., Lothe R. A., Aaltonen L. A., Pylkkänen L., Nyström-Lahti M., Seruca R., David L., Holm R., Ryberg D., Haugen A. (1993). Microsatellite instability is associated with tumors that characterize the hereditary non-polyposis colorectal carcinoma syndrome.. Cancer Res.

[OCR_01390] Perucho M., Peinado M. A., Ionov Y., Casares S., Malkhosyan S., Stanbridge E. (1994). Defects in replication fidelity of simple repeated sequences reveal a new mutator mechanism for oncogenesis.. Cold Spring Harb Symp Quant Biol.

[OCR_01396] Perucho M., Welsh J., Peinado M. A., Ionov Y., McClelland M. (1995). Fingerprinting of DNA and RNA by arbitrarily primed polymerase chain reaction: applications in cancer research.. Methods Enzymol.

[OCR_01401] Rhoer-Moja S., Cohen-Haguenauer O., Jouve C., Healy J. C., Vindimian M. (1993). Detection of quantitative polymerase chain reaction products by hybridization on magnetic support with 125I-radiolabeled probes: quantification of c-myc copy numbers.. Anal Biochem.

[OCR_01407] Rothberg P. G., Spandorfer J. M., Erisman M. D., Staroscik R. N., Sears H. F., Petersen R. O., Astrin S. M. (1985). Evidence that c-myc expression defines two genetically distinct forms of colorectal adenocarcinoma.. Br J Cancer.

[OCR_01415] Sato K., Miyahara M., Saito T., Kobayashi M. (1994). c-myc mRNA overexpression is associated with lymph node metastasis in colorectal cancer.. Eur J Cancer.

[OCR_01420] Sestini R., Orlando C., Zentilin L., Lami D., Gelmini S., Pinzani P., Giacca M., Pazzagli M. (1995). Gene amplification for c-erbB-2, c-myc, epidermal growth factor receptor, int-2, and N-myc measured by quantitative PCR with a multiple competitor template.. Clin Chem.

[OCR_01426] Shaw P., Bovey R., Tardy S., Sahli R., Sordat B., Costa J. (1992). Induction of apoptosis by wild-type p53 in a human colon tumor-derived cell line.. Proc Natl Acad Sci U S A.

[OCR_01431] Shibata D., Peinado M. A., Ionov Y., Malkhosyan S., Perucho M. (1994). Genomic instability in repeated sequences is an early somatic event in colorectal tumorigenesis that persists after transformation.. Nat Genet.

[OCR_01436] Shibuya M., Yokota J., Ueyama Y. (1985). Amplification and expression of a cellular oncogene (c-myc) in human gastric adenocarcinoma cells.. Mol Cell Biol.

[OCR_01441] Smith D. R., Myint T., Goh H. S. (1993). Over-expression of the c-myc proto-oncogene in colorectal carcinoma.. Br J Cancer.

[OCR_01445] Stewart B. W. (1994). Mechanisms of apoptosis: integration of genetic, biochemical, and cellular indicators.. J Natl Cancer Inst.

[OCR_01449] Stone B., Wharton W. (1994). Targeted RNA fingerprinting: the cloning of differentially-expressed cDNA fragments enriched for members of the zinc finger gene family.. Nucleic Acids Res.

[OCR_01454] Sugimoto T., Tsukamato F., Fujita M., Takai S. (1994). Ki-ras and c-myc oncogene expression measured by coamplification polymerase chain reaction.. Biochem Biophys Res Commun.

[OCR_01459] Thibodeau S. N., Bren G., Schaid D. (1993). Microsatellite instability in cancer of the proximal colon.. Science.

[OCR_01463] Untawale S., Blick M. (1988). Oncogene expression in adenocarcinomas of the colon and in colon tumor-derived cell lines.. Anticancer Res.

[OCR_01467] Wang J., Li L., Li S., Cui H., Shen G. (1994). A study of c-myc oncogene expression and amplification in colorectal cancer.. Chin Med Sci J.

[OCR_01471] Watson P. H., Safneck J. R., Le K., Dubik D., Shiu R. P. (1993). Relationship of c-myc amplification to progression of breast cancer from in situ to invasive tumor and lymph node metastasis.. J Natl Cancer Inst.

[OCR_01476] Welsh J., McClelland M. (1990). Fingerprinting genomes using PCR with arbitrary primers.. Nucleic Acids Res.

[OCR_01480] Yasuda J., Navarro J. M., Malkhosyan S., Velazquez A., Arribas R., Sekiya T., Perucho M. (1996). Chromosomal assignment of human DNA fingerprint sequences by simultaneous hybridization to arbitrarily primed PCR products from human/rodent monochromosome cell hybrids.. Genomics.

[OCR_01487] Yin Y., Tainsky M. A., Bischoff F. Z., Strong L. C., Wahl G. M. (1992). Wild-type p53 restores cell cycle control and inhibits gene amplification in cells with mutant p53 alleles.. Cell.

[OCR_01492] Yokota J., Tsunetsugu-Yokota Y., Battifora H., Le Fevre C., Cline M. J. (1986). Alterations of myc, myb, and rasHa proto-oncogenes in cancers are frequent and show clinical correlation.. Science.

[OCR_01497] Yonish-Rouach E., Resnitzky D., Lotem J., Sachs L., Kimchi A., Oren M. (1991). Wild-type p53 induces apoptosis of myeloid leukaemic cells that is inhibited by interleukin-6.. Nature.

